# Context counts: Investigating pain management interventions in HIV-positive men living in a rural area

**DOI:** 10.4102/phcfm.v15i1.3678

**Published:** 2023-05-09

**Authors:** Cameron Reardon, Antonia Wadley, Romy Parker

**Affiliations:** 1 Division of Physiotherapy, Department of Health and Rehabilitation Sciences, Faculty of Health Sciences, University of Cape Town, Cape Town, South Africa; 2 Ukwanda Centre for Rural Health, Department of Global Health, Faculty of Medicine and Health Sciences, Stellenbosch University, Cape Town, South Africa; 3 Division of Physiotherapy, Department of Health and Rehabilitation Sciences, Faculty of Medicine and Health Sciences, Stellenbosch University, Cape Town, South Africa; 4 Brain Function Research Group, School of Physiology, Faculty of Health Sciences, University of the Witwatersrand, Johannesburg, South Africa; 5 Department of Anaesthesia and Perioperative Medicine, Faculty of Health Sciences, University of Cape Town, Cape Town, South Africa

**Keywords:** primary health care, rural, pain, exercise, education, therapeutic relationship, self-efficacy

## Abstract

**Background:**

Pain remains a prevalent and burdensome complaint for people living with human immunodeficiency virus and/or aquired immunodeficiency syndrome (LWHA). Positive Living (PL), a multimodal pain intervention, reduced pain in female South Africans LWHA. We investigated the efficacy of the PL programme in South African males living with human immunodeficiency virus and/or acquired immunodeficiency syndrome (MLWA) in a rural community.

**Aim:**

To determine the effects of a multimodal pain intervention in MLWHA.

**Setting:**

Various primary care clinics in Manguzi, Kwa-Zulu Natal, South Africa.

**Methodology:**

Therapeutic relationship (TR) intervention alone or in combination with the PL programme were allocated to HIV-positive men between the ages of 18–40. Pain intensity and interference were the primary outcome measures. Secondary outcome measures included physical function, health-related quality of life, depressive symptoms and self-efficacy.

**Results:**

Forty-seven men (mean age 35 ± 3 years) were recruited with baseline mean pain severity of 5.02 (± 3.01) and pain interference of 4.6 (± 3.18). Nineteen men were allocated to the TR intervention alone, 28 were allocated to the TR intervention and PL programme. Attendance at the intervention sessions varied from 10% to 36%. No changes in any outcomes were recorded.

**Conclusion:**

Poor attendance at the intervention and follow-up sessions make these results an unreliable reflection of the intervention. Contextual factors including internal migration and issues around employment were identified. These may influence healthcare utilisation for MLWHA living in rural settings.

**Contribution:**

Unmet healthcare needs of MLWHA in a rural community have been identified. If we are to ‘leave no one behind’, healthcare interventions should account for context and be ‘rural-proofed’.

## Introduction

Uncontrolled pain has a detrimental effect on the health-related quality of life (HRQoL) of persons living with human immunodeficiency virus and/or aquired immunodeficiency syndrome (PLWHA) and is one of the most prevalent and burdensome symptoms associated with human immunodeficiency virus (HIV) and/or acquired immunodeficiency syndrome (AIDS).^1^ Pain as a biopsychosocial experience is well described,^1,2,3^ as is the fact that it should be addressed as such.^3,4^ It holds then that in the modern treatment era, comprehensive HIV and AIDS care delivered at a primary care level should adequately address the problem of pain holistically.

By providing services that include voluntary counselling and testing, provision of antiretroviral therapy (ART) and ongoing surveillance and monitoring to the most affected communities, great strides have been made in controlling the HIV and AIDS epidemic in the South African context.^5,6,7^ Primary care services for PLWHA have, however, not completely transformed. Clinical focus has not shifted beyond disease control towards optimising the quality of life of PLWHA.

Inadequate staffing and a lack of resources are but a few of the challenges that have contributed to an overburdened healthcare system in South Africa. This significantly limits its responsiveness to ongoing community needs.^8^ Future expansion of the package of services offered at a primary care level to include holistic pain management strategies for PLWHA must consider these challenges. In this regard, the Positive Living (PL) programme, which was developed for use in PLWHA living in under-resourced urban contexts, aims to actively engage communities in their own care through a peer-led delivery model. Utilising various components – education, exercise and cognitive behavioural strategies – the programme aims to develop self-management skills for effective pain management over a 6-week period.^4^

Encouraging results have been demonstrated for the PL programme. In a cohort of amaXhosa females living with human immunodeficiency virus and acquired immunodeficiency syndrome (LWHA) who participated in the 6-week intervention, significant improvements in pain intensity and pain interference, which could not be attributed to usual care, were noted.^9^ It was only following participation in the PL programme that a group of women showed improvements in their pain despite having undergone usual care for the 15 months preceding the programme. In that investigation, improvements were not limited to participants in the PL programme only. Similar improvements across outcomes of interest were present in women who simply received a workbook of educational content that covered similar material to that of the PL programme. The extent to which participants in this group engaged with the material was indeterminable and raised important questions regarding the mechanism by which pain improved – whether as a result of self-directed engagement or a ‘non-specific’ treatment effect. One explanation offered by the study authors is a ‘care effect’ – the analgesic response that may result as a direct consequence of a caring therapeutic relationship (TR) that may have developed over the course of the study between the participants and the researcher or researchers.^9^ By contrasting the environment created within the research study with what is typical for a primary health care setting, Parker et al. (2016) suggest a number of ways that a care effect may have been generated in their investigation. Specifically, they highlight the relatability of their research assistant (RA) who was able to communicate in participants’ first language; the frequency of contact between participants and the RA at follow-up sessions and the continuity of contact with the same RA at these sessions as important contributors to the development of a care effect. Recently, more supportive evidence for the beneficial effect of a TR on pain in rural amaXhosa women LWHA has emerged where similar measures were employed to facilitate a care effect.^10^ Facilitating a care effect by utilising measures that include improving communication and empathetic care is consistent with what has been widely described in the literature concerning the therapeutic alliance and physical health.^11^ Whether as a result of self-directed engagement with the workbook or in response to a continuous, caring relationship, these improvements in pain across cohorts of PLWHA are encouraging.

Understanding the underlying mechanisms by which pain and its secondary correlates including physical function, psychological wellbeing and HRQoL can be improved is of importance as it has the potential to influence health service delivery models for comprehensive HIV and AIDS care in overburdened health systems. Health service delivery models could either change to use task-shifting, by employing models of care that engage communities more actively in the provision of care, through peer-led groups or adapt simply by facilitating consistent and caring relationships between PLWHA and healthcare practitioners (HCP). It is also important to determine to what extent these improvements in pain reported by amaXhosa women in urban and rural contexts are applicable to other groups.

The potential impact that these interventions may have in rural settings where access to care is complicated and barriers are plentiful is promising. Rural settings bear much of the burden of HIV and AIDS. With a significant proportion of South Africa’s population residing in these settings (more than 30%),^12^ attention must be paid to these affected communities.

In this investigation, we aimed to determine whether the PL programme and a TR intervention, delivered at the primary care level, are clinically effective interventions for MLWHA residing in a rural community. The setting for this investigation was Manguzi situated in the Umkhanyakude district municipality, a municipality with a rural designation in the far northern region of Kwa-Zulu Natal, South Africa. Within the setting, HIV and AIDS is highly prevalent and is complicated by socioeconomic conditions including low levels of household income (< R3000.00 per month), low levels of education and high unemployment rates (> 45%).^13,14^

## Research methods and design

### Experimental design and participants

This study utilised a single-blinded experimental design with the mixed allocation of participants. Men living with HIV and/or AIDS residing in the rural community of Manguzi, South Africa were recruited. Participants were recruited through the HIV and AIDS and Sexually Transmitted Diseases and Tuberculosis (HAST) clinics at Manguzi Hospital and six primary-level clinics: Maputa, Thengani, Mshudu, Mahlangulu, Kwandaba and Bhekabantu. Recruitment strategies included screening of clinic waiting rooms, referrals from healthcare providers servicing these clinics, telephonic screening from available clinic records, as well as outreach screening through community health workers where telephonic contact was not possible.

Men were invited to participate if they were: HIV positive, between the ages of 18 and 40, on a stable ART regimen for a minimum of 6 months and had chronic pain and were first language isiZulu speakers. The designated age group was utilised as it encompasses the portion of the male population most affected by the HIV epidemic. Those above the age of 40 years were not considered for participation because of the increasing likelihood of multi-morbidity and the potential influence on baseline function and response to treatment. Participation was restricted to participants of a similar age in a similar phase of life in order to foster treatment efficacy in group environments. Chronic pain was defined as pain for at least the preceding 3 months^15^ and was determined by asking individuals a screening question from the Brief Pain Inventory (BPI) with the period of recall of the presence of ‘unusual pain’ adapted from 2 weeks to 3 months.^16^ Participants were excluded if they had previously participated in a biopsychosocial pain management programme if they did not meet the criteria of the American College of Sports Medicine guidelines for safe exercise^17^ and/or were noted to have cognitive impairment as evidenced from the available clinical records.

### Sample size

The primary outcome for this study was pain severity and interference. Sample size was calculated using the mean and standard deviation in pain severity scores (PSS), as measured by the BPI in a previous study of South African women LWHA, which reported standard deviations of 1.6, 2 and 3 for their different study groups.^8^ A sample size calculation is influenced by the spread of the data rather than actual values. We used a standard deviation of three points in our calculation, a conservative approach to allow for variations in pain report between men and women. This conservative approach required 48 participants to detect a minimum clinically significant change in PSS of three points^18^ with 90% power with statistical significance set at *p* < 0.05.

### Measures

Participants with limited literacy skills were guided through self-report questionnaires by the RA.

#### Sociodemographic and clinical characteristics

Sociodemographic and clinical data were collected from all participants at baseline and were collected from available health records. Sociodemographic characteristics included age, educational attainment and employment status. Clinical characteristics collected included HIV staging, CD4+ T-cell count, length of treatment, opportunistic infections and co-morbid conditions.

#### Pain severity and interference

The primary outcomes were pain severity and pain interference as measured by the isiZulu version of the BPI.^16^ The precursory version of the isiZulu tool, the Wisconsin Brief Pain Questionnaire has been validated in an amaZulu population.^19^ The BPI is a self-report tool, which primarily measures pain severity and pain interference. Pain severity was scored across three domains, ‘worst pain’, ‘least pain’ and ‘pain right now’ on an 11-point numerical rating scale (NRS) from 0 (no pain) to 10 (maximal pain). The responses were tallied, and a mean PSS (0–10) was generated. Similarly, pain interference was measured on an 11-point NRS and scored across seven domains resulting in a mean pain interference score (0–10). The seven domains include pain interference with ‘general activity’, ‘mood’, ‘walking ability’, ‘normal work’, ‘relations with other people’, ‘sleep’ and ‘enjoyment of life’.^16^

#### Secondary outcome measures

Health-related quality of life was measured on the EQ-5D-3L tool,^20^ a self-reported questionnaire that measures five dimensions: mobility, self-care, usual activities, pain and/or discomfort and anxiety and/or depression. We used the 3L version meaning that functioning was recorded in three categories, from poor function to better function. Responses from those questions are converted into a weighted health state index and range from zero to one with a higher score indicative of better health. The EQ-5D-3L also includes a visual analogue scale (VAS) where participants rate their perceived level of health from zero (‘worst imaginable health’) to 100 (‘best imaginable health’). The EQ-5D-3L is translated and available in isiZulu.^21^ The EQ-5D-3L tool has wide cross-cultural validity and has been validated across a number of Bantu languages.^22,23^

Physical function was measured with the Simmonds battery of tests, a physical performance bundle utilised in HIV and AIDS research.^24^ Physical performance tests include a 15 m preferred speed and fastest walk test, a six minute walk test, unloaded and loaded forward reach tests, timed repeated reach and sit to stand tests, a sock test and a timed belt tie.^24^

Depression was measured using the Beck Depression Inventory (BDI).^25^ The BDI is a self-report 21-item questionnaire designed to measure the severity of depressive mood symptoms. Participants are presented with 21 groups of statements and are required to indicate which statement best describes their experience. Each response is tallied, and a cumulative score is generated with higher scores indicative of greater depressive symptoms.^25^

Self-efficacy, the belief in one’s ability to manage common symptoms associated with HIV (including pain), was measured on the Self Efficacy for Managing Chronic Disease 6-item (SE-6) scale.^26^ Participants are required to rate their confidence in performing certain activities relating to their disease, on an NRS from 0 (‘not confident at all’) to 10 (‘totally confident’) in order to generate a mean self-efficacy score.^26^

Both the BDI and SE-6 were translated into isiZulu through one-way translation carried out by an HCP, an occupational therapist working in mental health who is familiar with these constructs and resides in the research setting and is proficient in isiZulu and English. The translated tools were checked by the RA, and no interpretation issues were reported.

### Interventions

No control group was included in this study as evidence demonstrates that usual care does not result in improvements in pain in PLWHA.^9^

Participants recruited for the study were allocated to the intervention groups using varied methods. Initially, allocation was randomised. Participants recruited at three clinic sites, HAST, Maputa and Thengani clinics, were randomly allocated to receive the TR intervention only or the TR intervention and PL programme. Randomisation was achieved through random number sequence generation. The geographical distribution of additional sites necessitated a more practical approach, however, as access to health facilities was complicated by distance. Further allocation was through convenience. Participants recruited at Mshudu and Mahlangulu clinics were allocated to the TR intervention. Participants recruited at Kwandaba and Bhekabantu clinics were allocated to the TR intervention and PL programme.

#### Therapeutic relationship intervention

A TR was fostered for all participants in this study. This TR was purposely generated through frequent contact with the same RA. While these contact sessions coincided with data collection time points, they differed from traditional data collection practices. The RA was encouraged to initiate all data collection sessions in an informal and empathetic manner with a ‘whole-person’ focus before meaning that consultations were not limited to data collection solely but included the RA enquiring after the participant’s well-being more generally too. General enquiry into the well-being of participants was coupled with empathetic communication strategies during these sessions. The purpose of this approach was to cultivate an atmosphere of trust and comfort. If individuals were unable to attend follow-up sessions in person, continuity was maintained through a telephonic follow-up session.

#### Positive Living programme

In addition to the TR intervention, participants (*n* = 28) were allocated to the 6-week PL programme. The PL programme has been comprehensively described elsewhere.^4^ The intervention comprised peer-led, weekly group sessions over the course of 6 weeks. Group sessions lasted approximately 2 h and in each session participants covered topical educational content. The educational themes are outlined in Table 1^4^, which has been adapted from Parker et al. (2014).^4^ The primary aim of the programme was to develop participants’ self-efficacy with regard to improved self-management of common distressing experiences associated with living with HIV and/or AIDS and to promote physical function.

**TABLE 1:** Weekly summary of educational content in the Positive Living programme.

Week	Theme	Summary
Week 1	Self-management and exercise	What is meant by self-management?
		Self-management plans
		Action steps
		Exercise
		Types of exercise (strength and aerobic training)
		Steps to success with exercise
		An exercise routine
Week 2	Managing common symptoms of HIV and AIDS	Symptom management
		Action charts for common symptoms
Week 3	Stress management	What is stress?
		Managing stress
		Sleep
		Communication with your healthcare worker
		Relaxation skills
Week 4	Pain	Causes of pain in HIV and AIDS
		Pain self-management
Week 5	Eating well	Balanced nutrition
		Dealing with barriers to eating well
		Food safety
Week 6	Continuing as a successful self-manager	Action planning for the future
		Reflecting on changes

*Source:* Adapted from Parker R, Jelsma J, Stein D. The development of an intervention to manage pain in amaXhosa women living with HIV. S Afr J Physiother. 2014;70(1):13–17. https://doi.org/10.4102/sajp.v70i1.259

HIV, human immunodeficiency virus; AIDS, acquired immunodeficiency syndrome.

During the sessions, participants also took part in a graded exercise intervention utilising a multimodal exercise programme, comprising aerobic exercise, modified strength training and flexibility exercises, which lasted between 20 min and 30 min. Upon completion of the exercises, participants were guided through progressive relaxation techniques including deep breathing and progressive muscle relaxation. Each session concluded with facilitated goal setting with the intention of translating learned skills into behavioural change.

### Procedure

Two local, amaZulu males, proficient in isiZulu and English who resided in the study context were recruited as RA’s to assist with recruitment and data collection processes, respectively. One RA was tasked with recruitment; the second was responsible for follow-up data collection. Data collection was a blind procedure. Preparatory training and ongoing support were provided by the investigator. A peer leader was identified from the local community through engagement with local healthcare professionals. In line with previous recommendations, the peer leader received over 40 h of formal training through an experiential learning model to facilitate knowledge transference and skill acquisition.^9^ Additional support was provided to the peer leader in the form of briefing and de-briefing sessions throughout the process to ensure fidelity.

Data were collected at weeks 0, 4, 8 and 24 for both groups. Data were collected through face-to-face contact sessions with telephonic data collection methods only utilised when face-to-face contact was not possible. Illiterate participants were guided through the data collection process by the RA.

Participants were reimbursed for travel expenses associated with participation in this study and airtime. Specifically, reimbursement was provided for the attendance of weekly PL intervention sessions as well as for data collection sessions.

### Statistical analysis

Data were determined to be normally distributed on the baseline primary outcome measure, using the Kolmogorov-Smirnov test. Parametric statistical analysis was performed. Descriptive statistics were used to describe the demographic data. Group differences for baseline, sociodemographic and clinical data were analysed using chi-squared (*χ*^2^) tests and *t*-tests. Multiple mixed model regression analysis was performed to determine the main effects for group and time and their interaction on primary and secondary outcome measures. This modelling was carried out by a statistician. Data were analysed according to an intention-to-treat methodology with missing data accounted for through regression analysis.

### Ethical considerations

Ethical approval was granted by the Human Research Ethics Committee (890/2014) at a large public university in the Western Cape and the National Department of Health (KZ 2015RP35751). The trial was registered with the Pan African Clinical Trials Registry (PACTR201410000902600).

## Results

**FIGURE 1:**
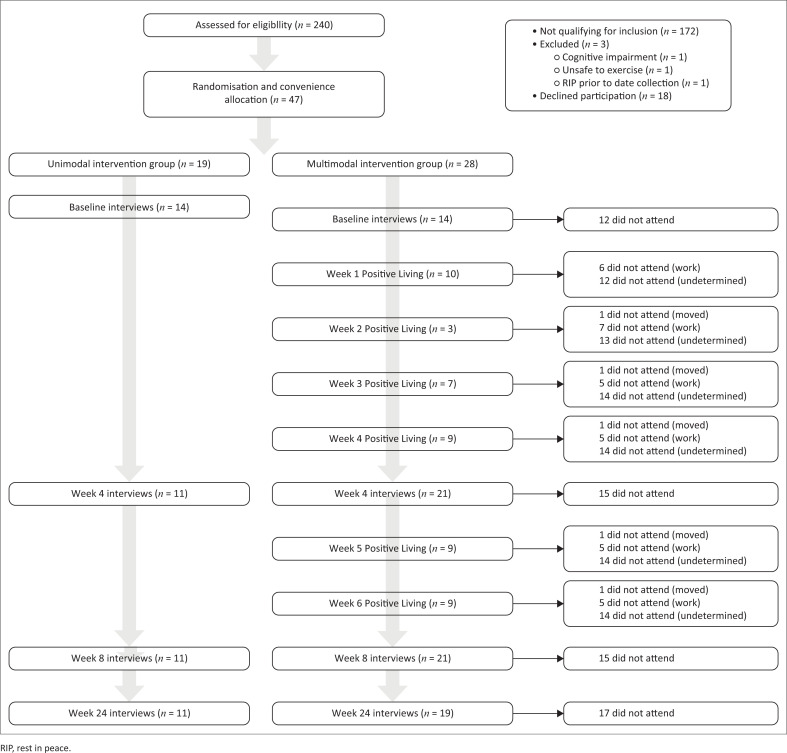
Experimental process.

Initially, 240 MLWHA were assessed for eligibility. Of those assessed, 172 MLWHA did not meet the inclusion criteria; 3 MLWHA were excluded from participation and 18 declined to participate. A total of 47 MLWHA were recruited for the study (see Figure 1).

### Sociodemographic and clinical characteristics

Because of the varied allocation methods, an unequal group allocation resulted with 28 participants assigned to the PL and TR group and 19 participants assigned to the TR group. Figure 1 illustrates the experimental process.

Table 2 shows the sociodemographic data and clinical characteristics for the cohort and groups. Participants in this study were all in their 30s. Educational levels were largely variable with some participants having no formal schooling. Consistent with demographic data in low socio-economic settings, high levels of unemployment were observed. No significant differences were observed for sociodemographic data or clinical variables between the two groups (Table 2). Similarly, no differences between groups existed for clinical variables (Table 2). Participants were classified across all stages according to the WHO classification, but the majority of participants were classified as HIV stage I. On average, participants had achieved an acceptable level of immunocompetence according to the latest CD4+ T-cell counts. Low levels of co-morbidities were observed in this cohort. While treatment duration varied among participants, on average participants had been stable on long-term management.

**TABLE 2:** Sociodemographic and clinical characteristics of participants.

Characteristic	All participants (*n* = 47)			PL programme (*n* = 28)			TR intervention alone (*n* = 19)			Significance test		
	Mean ± s.d.	*n*	%	Mean ± s.d.	*n*	%	Mean ± s.d.	*n*	%	*t*-test	*χ*^2^-test	*p*-value
**Age†**										−1.02	-	0.31
Years	35 ± 3	-	-	36 ± 3	-	-	35 ± 3	-	-	-	-	-
**Educational attainment†**										0.44	-	0.66
Years of schooling	9 ± 3	-	-	8 ± 3	-	-	9 ± 3	-	-	-	-	-
**Employment status**										-	4.01	0.68
Employed	-	-	-	-	-	-	-	-	-	-	-	-
Permanent employment	-	10	21	-	7	30	-	3	17	-	-	-
Temporary employment	-	6	13	-	4	15	-	2	11	-	-	-
Self-employment	-	5	11	-	4	15	-	1	5	-	-	-
Unemployed	-	24	51	-	12	42	-	12	63	-	-	-
Missing	-	2	4	-	1	4	-	1	5	-	-	-
**HIV stage**										-	1.27	0.74
Stage I	-	25	53	-	15	54	-	10	53	-	-	-
Stage II	-	7	15	-	4	14	-	3	16	-	-	-
Stage III	-	10	23	-	7	25	-	3	16	-	-	-
Stage IV	-	5	11	-	2	7	-	3	16	-	-	-
**CD4+ T-cell counts**												
Baseline CD4 at ART initiation (cells/µL)‡	205 ± 144	-	-	211 ± 138	-	-	194 ± 158	-	-	-0.34	-	0.74
Latest CD4 (cells/µL)†	415 ± 196	-	-	418 ± 203	-	-	411 ± 190	-	-	-0.12	-	0.91
**Length of treatment†**										-0.98	-	0.33
Months	41 ± 30.5	-	-	45 ± 33.6	-	-	36 ± 24.4	-	-	-	-	-
**Co-morbidities**										-	0.78	0.68
Hypertension	-	3	6	-	2	7	-	1	5	0	-	-
Epilepsy	-	1	2	-	1	4	-	0	0	-	-	-
None	-	43	91	-	25	89	-	18	95	-	-	-

PL, Positive Living; TR, therapeutic relationship; ART, antiretroviral therapy.

† , *t*(45);

‡ , *t*(35).

### Baseline measures

Baseline data for all primary and secondary outcomes are presented in Table 3 and Table 4. At baseline, this cohort presented with moderate pain severity and interference, moderately high HRQoL and fair self-efficacy but with mild depression.^14,15,16,17,18,19^

**TABLE 3:** Baseline outcome measure data for pain, HRQoL, depression and self-efficacy.

Outcome	All participants (*N* = 47)		PL programme (*N* = 28)		TR intervention (*N* = 19)		Reference values
	Mean	s.d.	Mean	s.d.	Mean	s.d.	
**Pain**							
PSS	5.02	± 3.01	5.33	± 3.20	4.55	± 2.73	-
PIS	4.65	± 3.18	5.01	± 3.02	4.11	± 3	-
Minimal	-	-	-	-	-	-	-
Mild	-	-	-	-	-	-	1 -3
Moderate	-	-	-	-	-	-	4 – 6
Severe	-	-	-	-	-	-	7 - 10
**HRQoL**							
EQ-5D-3L index score†	0.64	± 0.3	0.66	± 0.3	0.6	± 0.29	-
VAS‡	62.45	± 23.1	65.86	± 20.65	57.36	± 26.33	-
**Depression**							
BDI score	17.47	± 10.89	18.23	± 13.02	16.29	± 6.53	-
Minimal	-	-	-	-	-	-	0-13
Mild	-	-	-	-	-	-	14-19
Moderate	-	-	-	-	-	-	20-28
Severe	-	-	-	-	-	-	29-63
**Self-efficacy**	-	-	-	-	-	-	
SE-6 score§	6.39	± 2.2	6.02	± 2.26	6.93	± 2.06	-

† , 0 (a state as bad as being dead) – 1 (full health);

‡ , 0 (worst health imaginable) – 100 (best health imaginable);

§ , 0 (not at all confident) – 10 (totally confident).

**TABLE 4:** Baseline outcome measure data for Simmonds battery of tests.

Outcome	All participants (*n* = 47)		PL programme (*n* = 28)		TR intervention (*n* = 19)	
	Mean	s.d.	Mean	s.d.	Mean	s.d.
**Physical function**						
Preferred walking speed (m.s^−1^)	0.96	± 0.29	1.06	± 0.27	0.8	± 0.24
Fastest walking speed (m.s^−1^)	1.3	± 0.35	1.36	± 0.36	1.19	± 0.31
6-min walk test (m)	383.6	± 99.3	389.8	± 108	373.4	± 87.4
Timed repeated sit to stand (s)	4.10	± 1.62	4.37	± 1.93	3.64	± 0.82
Unloaded forward reach (cm)	101.24	± 14.5	110.11	± 17.13	110.45	± 9.47
Loaded forward reach (cm)	95.6	± 14.09	94.64	± 16.77	97.18	± 8.54
Timed repeated reach up (s)	6.22	± 2.48	6.58	± 2.83	5.65	± 1.76
Timed belt tie (s)	24.62	± 7.67	23.66	± 7.7	26.2	± 7.71
Timed sock test (s)	9.01	± 7.54	9.55	± 7.63	8.14	± 7.69

PL, Positive Living; TR, therapeutic relationship; m.s^−1^, metres per second; s, seconds.

### Change over time

**FIGURE 2:**
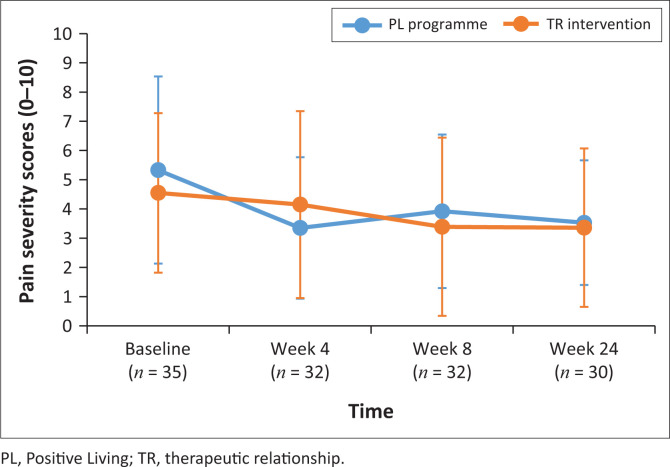
Pain severity scores over time.

**FIGURE 3:**
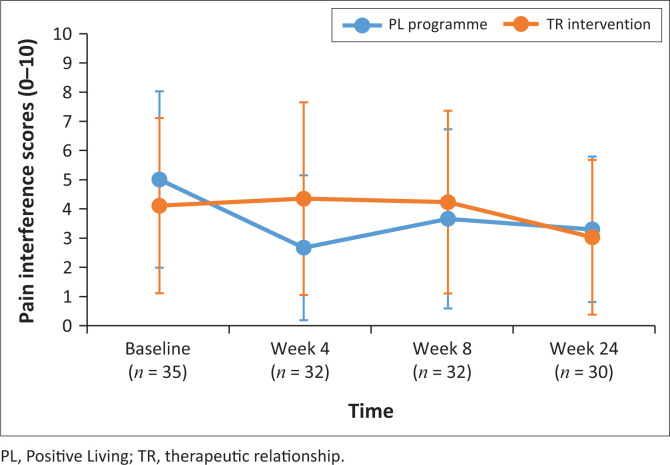
Pain interference scores over time.

Primary pain outcome measures of pain severity and pain intensity were monitored over the duration of the study. Following a cumulative mixed-model ordinal regression analysis, no significant change in pain severity and pain interference was observed for either group (*P* > 0.05). Pain severity scores and pain interference scores (PIS) are depicted in Figure 2 and Figure 3.

A similar lack of effect was observed across secondary outcome measures. Health-related quality of life as quantified by EQ-5D-3L index, and VAS scores showed no significant improvements over time for both groups. Physical function remained largely unchanged in both groups across most measurable categories. Beck depression inventory scores remained unchanged and no improvements in self-efficacy were observable for either group.

### Participation

**FIGURE 4:**
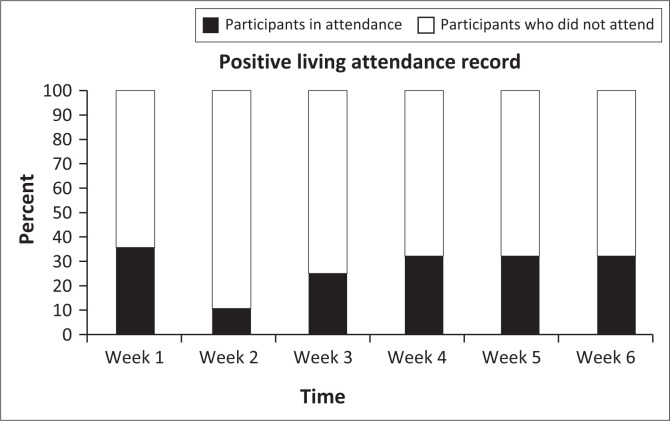
Participation in the Positive Living programme.

Participation in both the PL programme and TR interventions is illustrated in the experimental process diagram in Figure 1. High levels of non-participation were observed across both groups. Participation with the PL programme over the 6-week period is represented in Figure 4. Rates of participation peaked in the first week with 36% participation. Following a sharp decline in Week 2, participation rates plateaued somewhat for the remainder of the intervention period. Only nine participants (33%) attended three or more sessions and of those only two participants (7%) attended all sessions. Half of the participants allocated to this treatment arm did not attend a single intervention session.

Non-participation was observed at data collection points too, thereby limiting the development of a TR for all participants. Participation ranged from 63% to 74% across data collection time points.

Reasons for non-participation were not formally recorded. However, insofar as was possible, the RA followed up with participants telephonically and at the same time, enquired regarding non-participation. Feedback from the RA to the primary investigator revealed that participants’ reasons for non-participation were typically around internal migration and informal employment. This is evident too from participant responses for missed sessions for which data were recorded (see Figure 1). The RA reported that a proportion of participants across both cohorts had relocated during the investigation to more urban settings in pursuit of employment opportunities. For locally employed participants remaining in the area, participation was challenging. According to the RA, participants reported unsupportive attitudes of employers towards health-seeking behaviour and a reluctance to accept medical notes. Participants were also concerned that missed work would result in lost wages with one participant reporting an opportunity cost for seeking healthcare that would have financial implications.

Finally, the RA reported that for a small group of participants no clear reason was provided for their non-participation. The RA reported that when probed, some participants were evasive or gave incongruent responses about non-participation. This mistrust was encountered by the RA during telephonic recruitment and in person once participants had been recruited to the study. The RA’s interpretation of these encounters was that there was a general atmosphere of mistrust. It is possible that this mistrust could be an indicator of HIV stigma.

## Discussion

The aim of this study was to determine the efficacy of the PL programme and a TR intervention for pain in rural South African MLWHA. No improvements in pain severity, interference, HRQoL, physical function, depression or self-efficacy were seen over the 24 weeks of the study. The results may not be an accurate reflection of the efficacy of either intervention because of low rates of participation. The comments from the men about why they did not participate provide insight into social and logistical issues specific to MLWHA and pain in rural South Africa. These findings may speak to the contextual appropriateness of the interventions, particularly in rural settings and are worthy of further investigation.

These results are in contrast to a similar study in rural South African women.^10^ In that group of amaXhosa women living in a rural area in the Eastern Cape, all clinical outcomes improved in both the TR intervention and TR and PL intervention groups. Participation with the PL programme was better (38% – 65%) than observed in this cohort as was attendance at follow-up at data collection points (55% – 100%). Those findings suggest that the routine follow-up by a caring individual in the TR intervention improved outcomes in women LWHA. The findings are similar to those observed in the first study in another group of amaXhosa women in an urban setting.^9^ Not only did clinical outcomes improve in both the intervention and control groups, but there were reports of the women amassing social groups at their homes in order to work through the education book as a group.^9^ The efficacy and appropriateness of these interventions for men are called into question. Specific contextual factors that may have influenced participation included migration and mobility, informal employment and income insecurity and HIV-associated stigma.

### Migration and mobility

The effect of internal migration on participation in the present study was clearly articulated by participants through feedback obtained from the RA during this investigation. Some participants reported that they had moved during the study from their rural residences to surrounding urban areas. Subsequently, this limited either participation with the PL programme or the development of a TR or both.

Evidence from the most recently available census data suggests that 5% of South Africans migrated internally in the 5 years preceding the census.^27,28,29^ Internal migration is a dynamic phenomenon and may take on many forms, one of which is the temporary movement of the population between rural and urban areas, often primarily to access economic opportunity and employment.^30^ This phenomenon has its roots in the racially segregationist policies and discriminatory migration and urbanisation controls imposed by the apartheid government resulting in a legacy of spatial inequality.^29^ Despite changes in legislation and policy in the post-apartheid era, high rates of rural-urban migration have persisted.^27,28^ Indeed, temporary migration actually increased following the end of apartheid.^31^ While women are more mobile than previously, men still appear to be the most mobile.^28^

It is recognised that internal migration impacts HIV care, specifically through its effect on the spread and transmission of HIV.^32^ To our knowledge, this work is the first to suggest the potential barrier that internal migration practices may pose to pain management services for PLWHA residing in rural areas.

Considering the high levels of internal migration within this population group, health system rather than individual recommendations for care may be most appropriate. In this specific context, a strong, cohesive, systemic response that promotes continuity of care across different service points seems to be an important priority. Continuity of care as a construct is broad and beyond relational continuity encompasses additional aspects like informational and managerial continuity.^33^ While these three aspects of continuity: relational, informational and managerial continuity are linked, evidence suggests individual factors influence their relative importance to patients.^34^ In a highly mobile population, where relational continuity with HCPs may not be possible, informational and managerial continuity may be more important.

Informational continuity could be achieved through consistent cross-boundary and inter-facility communication facilitating the exchange of clinical information and access to medical records. While it is standard practice for PLWHA to be provided with referral letters to facilitate the exchange of clinical information between service points, the clinical information contained therein focuses solely on disease control. The absence of any HRQoL indicators in these clinical records is telling. This is concerning, particularly when we consider that patients with comorbid health conditions often under-report or fail to report symptoms such as pain in fear that they may distract the treating clinician from their primary complaint or be labelled as problematic.^35,36,37^ We assert that routine assessment for pain for PLWHA, a strategy that has been advocated for in other populations,^38^ is the first step to promoting adequate pain management in this population. However, simply screening patients for the presence of pain is unlikely to translate into better pain management. Rather, we recommend individualised, comprehensive, interprofessional and multidimensional assessment of PLWHA who present with persistent pain following routine screening. Additionally, we encourage the review of standard continuity of care records for PLWHA who are transferring care between service points and advocate for the inclusion of pain-related measures as well as measures of physical function and mental health within standard guidelines as a necessary step to promoting greater continuity of care.

### Informal employment and income insecurity

For those participants unaffected by internal migration, non-participation seemed to be explained, in part, by informal employment and income insecurity. Anecdotally, many of the participants who remained within the study context for the duration of the investigation were employed in the informal sector. The influence of the informal sector on health care utilisation is important as a high number of South Africans are informally employed. Furthermore, that informal employment poses challenges to the achievement of universal access to HIV prevention, treatment, care and support is well accepted.^39^ In particular, the informal sector’s reliance on a small labour base, the lack of access to health facilities and social protection available to informal workers, as well as the fragility of informal workers’ livelihoods, are all factors that may influence the utilisation of health services.^40^ Primarily, the opportunity cost of missed work and associated financial loss that would have resulted from participation with the intervention was a concern for study participants. The lack of social protection afforded to those employed in the informal sector, and the subsequent impact on healthcare utilisation highlights the need for intersectoral collaboration around healthcare issues, particularly for PLWHA.

The impact of informal employment on participation in medical research within rural contexts needs to be considered. One area for consideration is that of reimbursement. In this study, participants were compensated for participation in a manner that most closely reflects the reimbursement model – a revenue neutral model in which participants were reimbursed for any expenses incurred as a result of participation.^41^ An alternative strategy that could be considered is that of the wage model – where participants are compensated for participation in accordance with the accepted scale of unskilled and essential jobs.^41^ However, there are significant concerns associated with this alternative approach in terms of long-term sustainability and undue influence, including skewing of the data and recruitments that are not truly representative of the study population.^42^ In the absence of evidence-based recommendations, compensation for medical research participation in a rural setting, where participants may be particularly vulnerable to undue influencing, potentially resulting in a recruitment bias requires consideration.

While the influence of internal migration and informal employment and/or income insecurity on participation is considerable, it does not entirely explain the rates of non-participation in this cohort, particularly for unemployed participants who remained within the setting for the duration of the study. For these participants, there seem to be additional factors that may have influenced their participation, but these are less clear. One potential mechanism for non-participation in this cohort may have resulted from HIV-associated stigma.

### HIV-associated stigma

Anticipated stigma has been shown to relate to poor treatment compliance and clinic service utilisation in PLWHA.^43,44^ Anticipated stigma refers to the degree to which PLWHA believe that they may be subjected to prejudice or discriminated against because of their health status.^45^ HIV-associated stigma remains a highly prevalent issue in the South African context with local data demonstrating high rates of perceived stigma^46^ and discrimination following disclosure.^47^ Understandably, this is likely to result in an unwillingness to disclose on the part of PLWHA. Data suggest that for men living with HIV and/or AIDS simply seeking care regularly at a clinic may increase the risk of status exposure.^43^ The phenomenon whereby simply being seen regularly at a clinic may result in inadvertent disclosure has been termed forced disclosure and refers to the unintentional but overt identification of PLWHA in healthcare settings.^48^ In order to avoid forced disclosure, PLWHA often access HIV care under the guise of some other disorder.^43^ Comparatively, women appear to have more ‘reasons’ for accessing health care than their male counterparts and as such access to care for men may be more problematic.^43^ It could be that for this cohort attending clinic regularly to participate in an intervention may confer a risk for forced disclosure in a small rural community. Additionally, simply by participating in the intervention, one inadvertently discloses one’s status to other participating members. The perceived risk of *disclosure-through-participation* may be unacceptable to many PLWHA. Disclosure-through-participation and its relation to the study personnel also need to be considered in this setting. Both RAs and the peer leader for the PL intervention were local community members. The engagement of participants with study personnel was another potential source of forced disclosure and may have influenced recruitment and retention. Conducting research and interventions for PLWHA out of the clinic setting should be considered in the future. It is also clear that stigma reduction interventions are necessary to complement standard HIV and AIDS care and to promote healthcare utilisation. For PLWHA, pain cannot be managed in isolation.

While this work highlights the importance of contextual factors that may influence participation in medical interventions for MLWHA in a rural setting, we are unable to determine with certainty the extent to which each of these variables directly influenced participation in this research. When considering these findings in the context of what is known about broader issues of access to health care for rural communities, these findings are not surprising. Nevertheless, we recommend ongoing focussed research to explore contextual barriers to care for rural communities in order to inform the development of inclusive healthcare strategies that ‘leave no-one behind’^49^ – what some have called ‘rural proofing’.^50^

## Conclusion

Despite the intuitive appeal of a biopsychosocial intervention for pain delivered at a primary care level and the fact that the intervention has been previously successful in a cohort of rural South African women, we were unable to demonstrate any benefit from our intervention in rural South African men. The lack of treatment response observed was likely to have resulted from poor participation in both arms of the study. This poor participation possibly resulted from a myriad of sociocultural and economic factors including internal migration, informal employment and HIV-associated stigma. Contextually, relevant solutions are necessary to overcome these barriers to care. To this end, we propose that solutions, whether research or clinically oriented, are developed through participatory engagement with affected communities so that context is accounted for in the design of interventions.
